# Antihypernociceptive, Anxiolytic, and Antidepressant Properties of Aqueous and Ethanol Extracts of *Dissotis thollonii* Cogn. (Melastomataceae) in Mice

**DOI:** 10.1155/2020/8886894

**Published:** 2020-09-21

**Authors:** Stephanie Flore Djuichou Nguemnang, Eric Gonzal Tsafack, Marius Mbiantcha, Gilbert Ateufack, William Yousseu Nana, Albert Donatien Atsamo, Carine Flore Adjouzem, Vanessa Matah Marthe Mba, Egbe Ben Besong

**Affiliations:** ^1^Laboratory of Animal Physiology and Phytopharmacology, Department of Animal Biology, Faculty of Science, University of Dschang, P.O. Box 67 Dschang, Cameroon; ^2^Laboratory of Animal Physiology, Faculty of Science, University of Yaounde I, P.O. Box 812, Yaoundé, Cameroon; ^3^Department of Zoology and Animal Physiology, Faculty of Science, University of Buea, P.O. Box 63, Buea, Cameroon

## Abstract

Diabetic neuropathy, which affects 7 to 9% of the world's population and that is usually accompanied by anxiety and depression, is chronic pain that results from impaired function of the central or peripheral nervous system. This study aimed at evaluating the antihypernociceptive, antiallodynic, anxiolytic, and antidepressant effects of *Dissotis thollonii* extracts. Diabetic neuropathy was induced by intraperitoneal injection of streptozotocin (200 mg/kg) in mice. The aqueous and ethanol extracts (250 and 500 mg/kg) were administered orally. Hyperalgesia (thermal and chemical), allodynia (mechanical and thermal), anxiety (high plus labyrinth, light-dark box, and social interaction), and depression (open field test, suspension test tail, and forced swimming test) were evaluated, and then the levels of some cytokines and growth factors were determined. The aqueous and ethanol extracts of *Dissotis thollonii* demonstrated significant antihypernociceptive (inhibition of hyperalgesia and allodynia), anxiolytic, and antidepressant activities in mice made diabetic by STZ. The extracts also significantly inhibited (*p* < 0.001) the levels of TNF-*α*, IL-1*β*, and IL-6 in the blood as well as the levels of TNF-*α*, IL-1*β*, IL-6, IGF, and NGF in the sciatic nerve. This study shows that the extracts of *Dissotis thollonii* have antihypernociceptive and neuroprotective effects which could be linked to the inhibition of proinflammatory cytokines and growth factors in the blood and the sciatic nerve.

## 1. Introduction

Diabetes is one of the most common chronic medical conditions with several complications such as nephropathy, retinopathy, and neuropathy. Diabetic neuropathy is the most common and causes pain [1] which is defined as an alarm sign to protect the body when it is acute whereas in the chronic case, it becomes a disease [2]. Diabetes-induced deficits in motor and sensory nerve conduction velocities and other manifestations of diabetic peripheral neuropathy have been well correlated with chronic hyperglycemia. The first characteristic of diabetes mellitus is hyperglycemia, which plays a crucial role in the development and progression of diabetic neuropathy. Neuropathic pain is a form of chronic pain that results from damage or abnormal function of the central or peripheral nervous system. The main consequence of diabetic neuropathy is pain, and neuropatic pain is chronic pain characterized by hyperalgesia (sensitivity to harmful stimuli) and allodynia (abnormal pain sensitivity to previously painless stimuli) [3, 4]. The pathological mechanisms involved in diabetic neuropathy include microvascular damage, metabolic disorders, and changes in the interactions between the neuronal and immunological system alongside activation of glial cells. Several studies have reported that the neurotransmitter system and neurodegeneration can cause changes in pain perception in patients with diabetic neuropathy and that the mechanism of pathogenesis is not yet understood [5, 6]. Interleukin (IL-6 and IL-1*β*) and tumor necrosis factor (TNF-*α*) are the inflammatory markers that are elevated in hyperglycemia-induced neuropathic patients, implying that the inflammatory condition exists [7]. At the same time, IGF and NGF are the neuronal growth factors having the most important function in the survival and maintenance of the sympathetic and sensory nerves in hyperglycemia-induced neuropathic patients [8]. Neuropathic pain affects 7% to 9% of the world's population [4].

The streptozotocin-induced diabetes model is relatively simple, with a single injection either intraperitoneally or intravenously. Therefore, the diabetic rat induced by streptozotocin has been widely used to study the mechanism of diabetic neuropathic pain and to evaluate potential therapies [9]. Streptozotocin (2-deoxy-2 (3-methyl-3-nitrosoureido)-D-glucopyranose) is an analogue of nitrosourea. It is toxic to pancreatic *β*-cells secreting insulin by damaging its DNA [10, 11]. Animals treated with streptozotocin show behavioral signs of diabetic neuropathic pain, including a significant reduction in the withdrawal threshold at mechanical pressure and in the latency to the withdrawal of a harmful thermal stimulus [12]. Thermal hyperalgesia is an increased sensitivity to pain, which can be caused by damage to the nociceptors or peripheral nerves. In diabetic animals, the injection of formalin into the paw is characterized by a harmful biphasic behavior of trembling [13]. In people with diabetic neuropathy, there is a significant development of nephropathy, sexual dysfunction, and even cardiovascular complications, generally associated with the development of anxiety and depression [14, 15], hence the need to find a treatment capable of reducing pain in these people while exerting anxiolytic and antidepressant effects.

There are several treatment options for diabetic neuropathic pain, namely, pharmacological treatments (nonsteroidal anti-inflammatory drugs, tricyclic antidepressants, calcium channel ligand (pregabalin), opioid analgesics, tramadol, and lidocaine), transcutaneous nerve stimulation, neurosurgery, and alternative and behavioral treatments [16, 17], with pharmacological treatment as the dominant option for chronic pain [18]. All these treatments are limited for lack of effectiveness for some or for producing side effects for others, thus leading to incomplete remission in patients who are called upon to still live with pain [17, 19], hence the need to find more effective, safer treatments with no side effects and capable of targeting the underlying mechanism of pain [18, 20, 21]. This is why new therapies for the treatment of neuropathic pain are important with fewer or no side effects. Then, an alternative is the natural product of our environment, *D*. *thollonii*, which is a pantropical plant from the Melastomataceae family. It is traditionally used to treat sinusitis, typhoid fever, inflammatory diseases, and gastrointestinal disorders [22]. The plant has proven numerous activities such as antidiarrheic, antibacterial, antimicrobial, antioxidant, and antiulcerogenic activities [23–25]. In addition, Djuichou et al. [26] have shown anti-inflammatory and antiarthritic activities in vitro. All these activities are due to the presence of several metabolites and secondary compounds in the plant, in particular tannins, sterols, flavonoids, phenols, anthraquinones, and polyphenols [25, 26]. The aqueous and ethanol extracts of this plant significantly inhibit cell proliferation, the production of intracellular ROS, and the production of TNF-*α* in vitro [24]. Compounds such as arjunolic acid, betulinic acid, ellagic acid, 3, 3′-di-O-methylellagic acid 4′-O-*β*-D-xylopyranoside, *β*-sitosterol, 3, 3′-di-O-methylellagic acid, *β*-sitosterol-3-O-*β*-D-glucopyranoside, and *β*-sitosterol-3-O-D-glucopyranosyl-6′-mirystate have already been isolated from this plant [25]. Although this plant is widely used in traditional medicine to treat several pathologies of the organism, no scientific information to date has demonstrated its antihyperalgesic and neuropharmacological properties. In our continuous search for bioactive extracts of plants used in traditional Cameroonian medicine [27] and in order to support and improve the traditional use of *D*. *thollonii*, we were committed to carrying out this study on antihypernociceptive, anxiolytic, and antidepressant properties of extracts of leaves of *D*. *thollonii* on the model of diabetic neuropathy induced by injection of STZ in mice.

## 2. Materials and Methods

### 2.1. Plant Material and Preparation of Extracts

Samples of the plant leaves were collected in the city of Dschang (West Region, Cameroon) and then identified by comparison with another reference at the National Herbarium Yaounde (no. 133292/SRF Cam). Then, the freshly harvested leaves were dried under shade and crushed into a fine powder which was used for the preparation of the different extracts. For the aqueous extract, 500 g of powder was macerated (72 hours) in 500 ml of distilled water. After filtration (Whatman paper no. 4), the solution obtained was evaporated at 40°C to give 41 g of aqueous extract (yield 8.2%), while the same amount of powder was macerated in 500 ml of ethanol for 72 hours, filtered, and then concentrated in a rotary evaporator (96°C) to give 48 g of the ethanol extract (9.6%).

### 2.2. Experimental Animals

The Mus musculus Swiss strain mice (females and males) 6 to 8 weeks old and weighing between 20 and 30 g were used in this study. These were provided by the Research Institute of Chemistry, International Center for Chemical and Biological Sciences (ICCBS) at the University of Karachi (Pakistan). During a week of acclimatization (temperature of 22 ± 1°C and 50 to 80% humidity), with a cycle variation of 12 h, the animals freely consumed a standard diet for rodents and previously filtered water. The treatment of these animals was in agreement with the Institutional Committee for the Protection, Use, and Standardization of Animals (IACUC) of the “International Center for Chemical and Biological Sciences (ICCBS)” (Protocol no. 1209004), and the study protocols accepted by the ICCBS Ethics Committee were followed.

### 2.3. Induction of Diabetes by Streptozotocin

The induction of diabetes was carried out according to the protocol of Sanchez-Ramirez et al. [28]. Diabetes was induced in mice by injection of STZ (200 mg/kg, *i*.*p*.). Three days after the injection, the blood sugar level of the animals was measured by taking a drop of blood obtained from the tail and using the Accu-Chek Performa glucometer (Roche Diabetes Care, Inc., Indianapolis, USA). Animals with blood glucose ≥300 mg/dl were retained for testing.

### 2.4. Distribution and Treatment of Animals

Forty-two (42) mice were divided into 7 groups of 6 animals each: group 1 (neutral control) consisting of mice having received no STZ and no treatment, group 2 (negative control) consisting of diabetic mice treated with a mixture of distilled water/DMSO (5%), group 3 (positive control) consisting of diabetic mice treated with 30 mg/kg of pregabalin, groups 4 and 5 consisting of diabetic mice treated with the aqueous extract at the doses of 250 and 500 mg/kg, respectively, and groups 6 and 7 made up of diabetic mice treated with the ethanol extract at the respective doses of 250 and 500 mg/kg. All the treatments were administered orally 2 weeks after the injection of STZ and lasted 4 weeks.

### 2.5. Antinociceptive Activities of Extracts of *D*. *thollonii*

#### 2.5.1. Evaluation of the Effect of Extracts of *D*. *thollonii* on Mechanical Allodynia Induced by Von Frey

After an acclimatization time of 25 minutes in metal cages, a 0.4 g Von Frey filament was applied to the plant on the surface of the right paw of each animal; one application was represented 10% and each animal received 10 applications in total; then the number of applications to which the animal reacted (withdrawal of the paw or opening of the ears) was expressed as a percentage [29].

#### 2.5.2. Evaluation of the Effect of Extracts of *D*. *thollonii* on Thermal Hyperalgesia Induced by Hot Plate

A hot plate (54 ± 1°C) was used for this test with a stop time of 12 seconds. Each mouse was placed on the heating part of the plate and the time indicating the start of the animal's reaction (jump or licking of the paw) was recorded [30].

#### 2.5.3. Evaluation of the Effect of Extracts of *D*. *thollonii* on Cold Acetone-Induced Allodynia

The method described by Mbiantcha et al. [31] was used for this test. Each mouse received 5 drops of acetone (with an interval of 5 minutes between the different drops). The reaction of the animal materializing the cold allodynia (rapid withdrawal of the paw in contact with the drop) was considered when the animal made two withdrawals of its paw on the 5 applications.

#### 2.5.4. Evaluation of the Effect of Extracts of *D*. *thollonii* on Formalin-Induced Chemical Hyperalgesia

In this test, the protocol used was that described by Rocha-Gonzalez et al. [1] with some modifications. On the last day after the administration of the various treatments, 20 *μ*l of 2.5% formalin was injected under the plantar fascia of the left hind paw of the mice. Immediately after the formalin injection, each mouse was placed individually in a cage and observed for 60 minutes; time during which the paw licking time was counted. The percentage of inhibition (%*I*) during the first and second phases was calculated according to the following formula:

(1) $$\begin{array}{c} \%I=\frac{C-T}{C}\times 100, \end{array}$$

where *C* is the licking time of the control group and *T* is the licking time of the treated group.

#### 2.5.5. Evaluation of the Effects of Extracts of *D*. *thollonii* on Body Weight

The body weight of the mice was measured in grams (g) using a balance every day from the first day before the injection of STZ until day 42; then the values were expressed as variations per week.

#### 2.5.6. Assessment of the Effect of Extracts of *D*. *thollonii* on Blood Glucose Levels

Each week, a drop of blood taken from the tip of each animal's tail was placed on a strip connected to an Accu-Chek Performa glucometer (Roche Diabetes Care, Inc., Indianapolis, USA) to obtain the blood sugar level for each mouse.

#### 2.5.7. Collecting Samples

At the end of all the treatments (day 43), all the animals were anesthetized by inhalation of chloroform vapor, the abdomen was opened, and the blood was taken in dry tubes without anticoagulant by catheterization of the abdominal artery and then centrifuged at 3000 rpm (15 minutes), and the serum obtained was transferred to Eppendorf tubes and stored at 20°C. Then, the sciatic nerve was immediately removed, ground in PBS (0.1 g/1 ml), and centrifuged at 3000 rpm (15 minutes at 4°C), and the obtained supernatant was stored at −20°C. The levels of TNF-*α*, IL-1*β*, and IL-6 were evaluated in serum; also, the levels of TNF-*α*, IL-1*β*, IL-6, IGF, and NGF were evaluated in the homogenate of the sciatic nerve. All these parameters were evaluated by enzyme-linked immunosorbent assay using ELISA Sandwich tests according to the protocols described in the supplier's ELISA kit manual (Elabscience Biotechnology Inc., USA).

### 2.6. Neuropharmacological Activities of Extracts of *D*. *thollonii*

To evaluate the anxiolytic and antidepressant activities of *D*. *thollonii* extracts, 6 nondiabetic mice and 36 mice that made diabetic according to the same protocol as previously described were used. The Mus musculus Swiss strain mice (females and males) 6 to 8 weeks old and weighing between 20 and 30 g were used in this study. These were provided by the Animal House of Laboratory of Animal Physiology and Phytopharmacology, Department of Animal Biology, Faculty of Science, University of Dschang. The animals were distributed and treated as in the previous test, except that the animals in group 3 (positive control) were treated with diazepam (1 mg/kg) for the anxiolytic test and with fluoxetine (5 mg/kg) for the antidepressant test. All the treatments were administered orally 2 weeks after the injection of STZ and lasted 4 weeks.

The experimental procedures have been approved by the local ethics committee and are in accordance with the guidelines for the study of pain in awake animals, published by the NIH Publication no. 85–23, “Principles of Animal Protection,” “Laboratory,” and Study of Pain, Ministry of Scientific Research and Technology, which adopted the European Union Guidelines on Animal Care and Experimentation (EWC Council 86/609).

#### 2.6.1. Evaluation of the Anxiolytic Activity of Extracts of *D*. *thollonii*

To assess the anxiolytic activity of the extracts, the elevated labyrinth, light/dark box, and social interaction tests were used. All animals were observed for 6 minutes.

For the raised labyrinth test, the labyrinth consisting of 2 open arms and 2 closed arms connected by a central platform was raised to a height of 50 cm from the ground, and then, each animal was placed individually in the center of the labyrinth facing a closed arm, and the time spent in each arm was recorded [32].

A box (45 × 27 × 27 cm) separated into two compartments connected by an open space (7.5 × 7.5 cm) was used for testing the light/dark box. Each mouse was placed in the center of the light compartment, and the time spent in this compartment was recorded [33]. As for the social interaction test, the animals were first isolated for an hour before and then placed in an open box to observe the time taken to fight, bite, and lick the neck [33].

#### 2.6.2. Evaluation of the Antidepressant Activity of Extracts of *D*. *thollonii*

To assess the antidepressant activity of the extracts, tail suspension, open field, and forced swimming tests were used. All animals were observed for 6 minutes.

The mice were hung individually by the tail from the top of a cage using adhesive tape and the head directed towards the base at a distance of 10 cm from the base. The duration of immobility was considered and noted using a video device when the animals were completely stationary [34].

To explore the locomotion of animals, the open field test was carried out using the modified methodology of Yi et al. [35]. The animals were placed in a box where the base was divided into 4 equal squares with white stripes; 24 hours after the last treatment, each mouse was placed in the central region of the soil and the number of passages was recorded by a video device.

The protocol of Bhattmisra et al. [36] was modified and used for the forced swimming test. Each animal was placed in a container of water for 6 minutes, and the immobility time was recorded using a camera for the last 4 minutes. Stillness was taken into account when the animals floated in the water without moving and kept their heads above the water.

## 3. Statistical Analyses

All data are presented on average of 6 animals ± SEM. The differences between the groups were assessed by ANOVA (unidirectional and bidirectional) followed by the Bonferroni posttest, then by ANOVA (bidirectional) followed by the Tukey posttest. Significant differences were considered at *p* < 0.05.

## 4. Results

### 4.1. Antinociceptive Effects of Extracts of *D*. *thollonii*

#### 4.1.1. Effects of Extracts of *D*. *thollonii* on Mechanical (Von Frey) and Cold Allodynia (Acetone)

**Figure 1:**
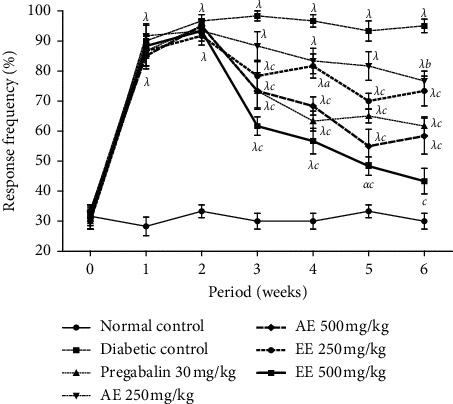
Effects of the aqueous (AE) and ethanol (EE) extracts of *Dissotis thollonii* on mechanical allodynia induced by Von Frey. Values are expressed as mean ± SEM for six animals and analyzed by two-way ANOVA followed by Bonferroni post hoc test. ^*α*^*p* < 0.05, ^*λ*^*p* < 0.001 when compared with the normal control and ^*a*^*p* < 0.05, ^*b*^*p* < 0.01, ^*c*^*p* < 0.001 when compared with the diabetic control.

The threshold for sensitivity to mechanical allodynia increased significantly (*p* < 0.001) during the first week in all animals in the untreated diabetic control group compared to the neutral control group (Figure 1). The administration of different extracts at different doses as well as pregabalin significantly (*p* < 0.001) reduced this threshold of sensitivity from the third week until the end of the experiment (sixth week). However, the ethanol extract was distinguished by its frequency of response very close to that of the neutral control at the sixth week.

**Figure 2:**
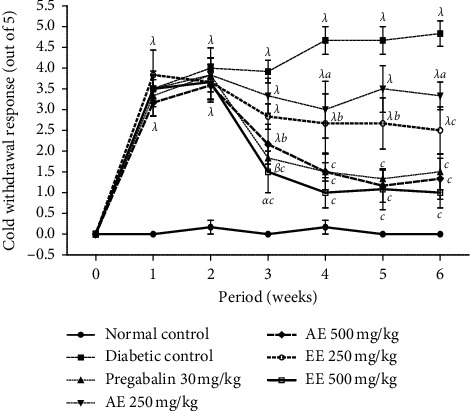
Effects of the aqueous (AE) and ethanol (EE) extracts of *Dissotis thollonii* on cold allodynia induced by acetone. Values are expressed as mean ± SEM for six animals and analyzed by two-way ANOVA followed by Bonferroni post hoc test. ^*α*^*p* < 0.05, ^*β*^*p* < 0.01, ^*λ*^*p* < 0.001 when compared with the normal control and ^*a*^*p* < 0.05, ^*b*^*p* < 0.01, ^*c*^*p* < 0.001 when compared with the diabetic control.

The variation of the response to the withdrawal of the leg of the animal caused by the cold (acetone) is presented in Figure 2. It appears from this figure that the withdrawal of the legs of diabetic animals significantly (*p* < 0.001) increased from the first week. During the third week, the two extracts at the dose of 500 mg/kg and pregabalin (30 mg/kg) significantly reduced the number of withdrawals of the animals' legs. This activity remained important and significant until the fourth week.

#### 4.1.2. Effects of Extracts of *D*. *thollonii* on Thermal (Hotplate) and Chemical (Formalin) Hyperalgesia

**Figure 3:**
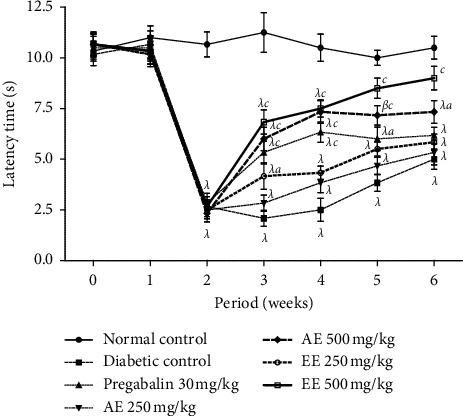
Effects of the aqueous (AE) and ethanol (EE) extracts of *Dissotis thollonii* on thermal hyperalgesia induced by hot plate. Values are expressed as mean ± SEM for six animals and analyzed by two-way ANOVA followed by Bonferroni post hoc test. ^*β*^*p* < 0.01, ^*λ*^*p* < 0.001 when compared with the normal control and ^*a*^*p* < 0.05, ^*c*^*p* < 0.001 when compared with the diabetic control.

The hot plate was used as a stimulus to assess the effects of the extracts on thermal hyperalgesia. The latency period for licking the paw in untreated diabetic animals was significantly (*p* < 0.001) reduced compared to that of animals in the neutral control group (Figure 3). From the third week, the increase in this latency period for licking the paw was significant (*p* < 0.05; *p* < 0.001) in animals treated with ethanol extract at doses of 250 mg/kg and 500 mg/kg of the two extracts, respectively, compared to the negative control. Only the 500 mg/kg dose of the either extract remained significant (*p* < 0.001; *p* < 0.05) until the last week.

**Figure 4:**
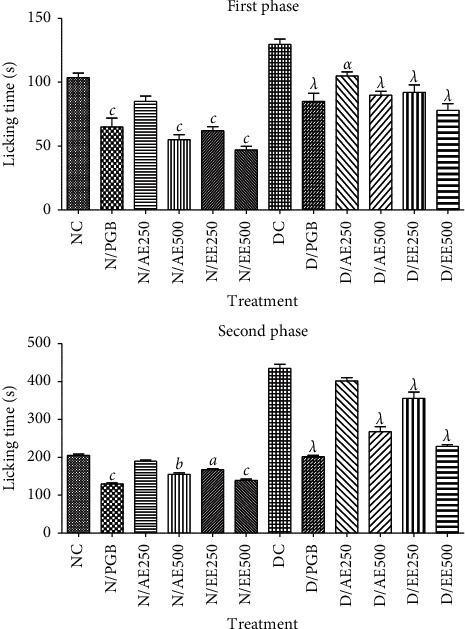
Effects of the aqueous (AE) and ethanol (EE) extracts of *Dissotis thollonii* on hyperalgesia induced by formalin. Values are expressed as mean ± SEM for six animals and analyzed by one-way ANOVA followed by the Tukey post hoc test. ^*α*^*p* < 0.05, ^*λ*^*p* < 0.001 when compared with the normal control and ^*a*^*p* < 0.05, ^*b*^*p* < 0.01, ^*c*^*p* < 0.001 when compared with the diabetic control.

After the formalin injection, a two-phase response was observed in nondiabetic mice as in their diabetic counterparts (Figure 4). The inhibitory effects of the extracts on formalin-induced chemical hyperalgesia were significantly observed in both nondiabetic and diabetic mice at all stages. Thus, at a dose of 500 mg/kg, the inhibitions were 46.95% (aqueous extract, first phase), 24.39% (aqueous extract, second phase), 54.66% (ethanol extract, first phase), and 32.20% (ethanol extract, second phase) in the nondiabetic mouse. For mice made diabetic, the inhibitions were 30.59% (500 mg/kg, aqueous extract, first phase), 39.85% (500 mg/kg, aqueous extract, second phase), 38.39% (500 mg/kg, ethanol extract, first phase), and 47.36% (500 mg/kg, ethanol extract, second phase). Pregabalin inhibited this hyperalgesia by 37.30% and 36.75%, respectively, for the first and second phases (nondiabetic mice) and then 34.45% and 53.64% in the first and second phase, respectively (diabetic mice).

#### 4.1.3. Effects of the Aqueous and Ethanol Extracts of *D*. *thollonii* on Blood Sugar and Body Weight

The glycemia increased significantly (*p* < 0.001) in all animals made diabetic from the first week compared to the animals in the neutral control group (Table 1). Extracts at all doses led to a drop in blood sugar in all diabetic animals, although this drop was not significant.

The body weight of all animals receiving streptozotocin decreased significantly (*p* < 0.001) from the first week of the experiment compared to the animals in the neutral control group (Table 2). This reduction in body weight was gradually corrected with the administration of the various extracts as well as pregabalin until the end of the treatment, although this effect of the treatment was not significant.

#### 4.1.4. Effects of the Aqueous and Ethanol Extracts of *D*. *thollonii* on Serum and Tissue Cytokine Levels

**Figure 5:**
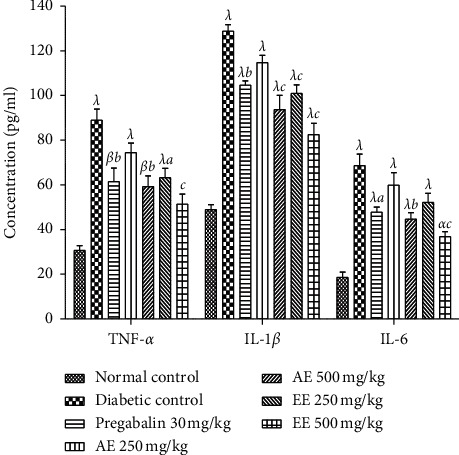
Effects of the aqueous (AE) and ethanol (EE) extracts of *Dissotis thollonii* on the levels of TNF-*α*, IL-1*β*, and IL-6 in the serum of diabetic mice. Values are expressed as mean ± SEM for six animals and analyzed by one-way ANOVA followed by the Tukey post hoc test. ^*α*^*p* < 0.05, ^*β*^*p* < 0.01, ^*λ*^*p* < 0.001 when compared with the normal control and ^*a*^*p* < 0.05, ^*b*^*p* < 0.01, ^*c*^*p* < 0.001 when compared with the diabetic control.

**Figure 6:**
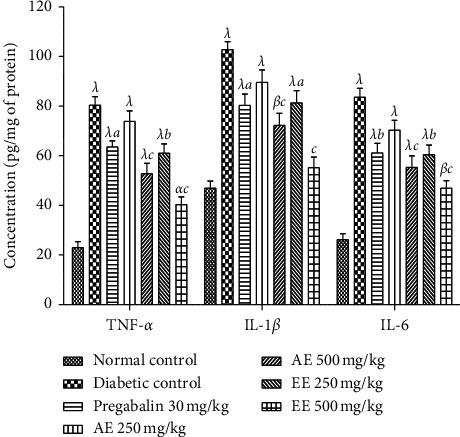
Effects of the aqueous (AE) and ethanol (EE) extracts of *Dissotis thollonii* on the levels of TNF-*α*, IL-1*β*, and IL-6 in the sciatic nerve of diabetic mice. Values are expressed as mean ± SEM for six animals and analyzed by one-way ANOVA followed by the Tukey post hoc test. ^*α*^*p* < 0.05, ^*β*^*p* < 0.01, ^*λ*^*p* < 0.001 when compared with the normal control and ^*a*^*p* < 0.05, ^*b*^*p* < 0.01, ^*c*^*p* < 0.001 when compared with the diabetic control.

The serum and tissue levels of cytokines (TNF-*α*, IL-1*β*, and IL-6) increased significantly (*p* < 0.001) in the serum of animals in the negative control group compared to those in the neutral control group (Figures 5 and 6). Treatment with aqueous and ethanol extracts (500 mg/kg) like pregabalin (30 mg/kg) resulted in a significant decrease in serum and tissue levels of TNF-*α* (*p* < 0.01), IL-1*β* (*p* < 0.001), and IL-6 (*p* < 0.01).

#### 4.1.5. Effects of the Aqueous and Ethanol Extracts of *D*. *thollonii* on Tissue Levels of IGF and NGF

**Figure 7:**
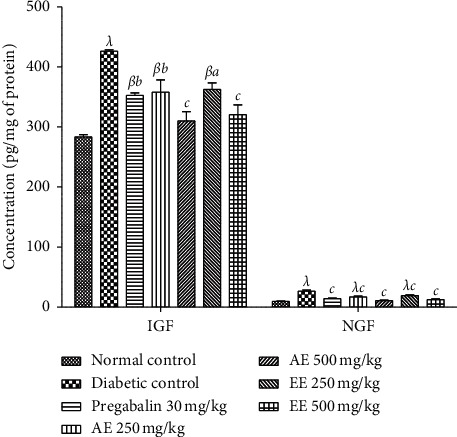
Effects of the aqueous (AE) and ethanol (EE) extracts of *Dissotis thollonii* on the levels of IGF and NGF in the sciatic nerve of diabetic mice. Values are expressed as mean ± SEM for six animals and analyzed by one-way ANOVA followed by the Tukey post hoc test. ^*β*^*p* < 0.01, ^*λ*^*p* < 0.001 when compared with the normal control and ^*a*^*p* < 0.05, ^*b*^*p* < 0.01, ^*c*^*p* < 0.001 when compared with the diabetic control.

Administration of streptozotocin in animals leads to a significant increase (*p* < 0.05; *p* < 0.01; *p* < 0.001) in IGF and NGF levels in the sciatic nerve in untreated diabetic animals compared to animals in the neutral control group (Figure 7). Both extracts at the dose of 500 mg/kg significantly (*p* < 0.01, *p* < 0.001) decreased the level of IGF compared to the negative control. As for the animals having received pregabalin, there was a significant (*p* < 0.001) decrease compared to the negative control. However, at the dose of 250 mg/kg, both extracts induced a significant effect on the level of IGF. The NGF level decreased significantly (*p* < 0.05; *p* < 0.001) compared to the negative control.

### 4.2. Neuropharmacological Effects of *D*. *thollonii* Extracts

#### 4.2.1. Anxiolytic Properties of Aqueous and Ethanol Extracts of *D*. *thollonii*

**Figure 8:**
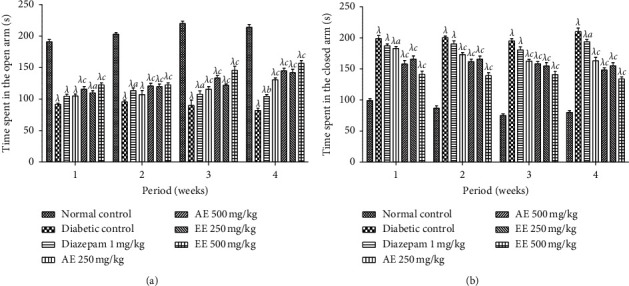
Effects of the aqueous (AE) and ethanol (EE) extracts of *Dissotis thollonii* on the absolute time spent in the open arms (a) and close arms (b) during 6 min of exposure to the elevated plus maze. Values are expressed as mean ± SEM for six animals and analyzed by two-way ANOVA followed by Bonferroni post hoc test. ^*λ*^*p* < 0.001 when compared with the normal control and ^*a*^*p* < 0.05, ^*b*^*p* < 0.01, ^*c*^*p* < 0.001 when compared with the diabetic control; s: seconds.

The results showing the behavior of the animals placed on the raised labyrinth are presented in Figure 8. It turns out that all the diabetic animals spent less time in the open arm and more time in the closed arm compared to the nondiabetic mice. The different treatments (aqueous extract, ethanol extract, and diazepam) reversed the trend by significantly increasing the time spent in the open and significantly reducing the time spent in the closed arms compared to animals in the negative control group.

**Figure 9:**
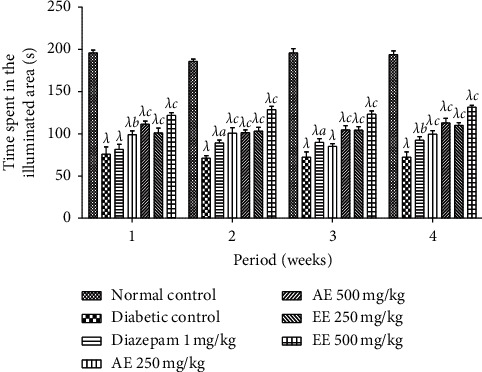
Effects of the aqueous (AE) and ethanol (EE) extracts of *Dissotis thollonii* on the absolute time spent in the open arms (light) compartment during 5 min of exposure to the light-dark box test. Values are expressed as mean ± SEM for six animals and analyzed by two-way ANOVA followed by Bonferroni post hoc test. ^*λ*^*p* < 0.001 when compared with the normal control and ^*a*^*p* < 0.05, ^*b*^*p* < 0.01, ^*c*^*p* < 0.001 when compared with the diabetic control; s: seconds.

The behavior of the animals placed in the box with two compartments (dark and light) is presented in Figure 9. We can see on this figure that the time spent in the lit area is significantly reduced in all diabetic animals compared to nondiabetic animals and that after the administration of the different extracts at the different doses and of diazepam, the time spent in the illuminated zone was significantly (*p* < 0.001; *p* < 0.05) increased compared to the negative control. This increase was gradual until the end of treatment (fourth week).

**Figure 10:**
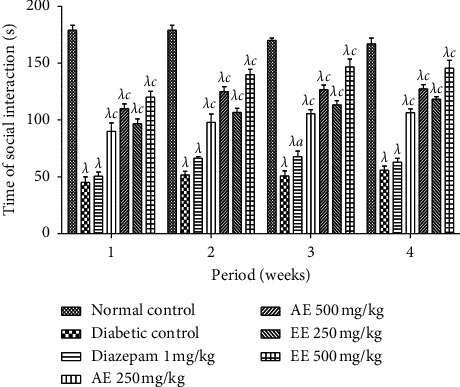
Effects of the aqueous (AE) and ethanol (EE) extracts of *Dissotis thollonii* on the absolute time spent for social interaction during 5 min. Values are expressed as mean ± SEM for six animals and analyzed by two-way ANOVA followed by Bonferroni post hoc test. ^*λ*^*p* < 0.001 when compared with the normal control and ^*a*^*p* < 0.05, ^*c*^*p* < 0.001 when compared with the diabetic control; s seconds.

The result of the social interaction test is presented in Figure 10. It can be seen that in the first week, the duration of the social interaction increased significantly (*p* < 0.001) in the animals which received the two extracts at the two doses, compared to the negative control. It should also be noted that this increase was gradual until the end of treatment. The positive control significantly increased the duration of social interaction only at the second week of treatment.

#### 4.2.2. Antidepressant Properties of the Aqueous and Ethanol Extracts of *D*. *thollonii*

**Figure 11:**
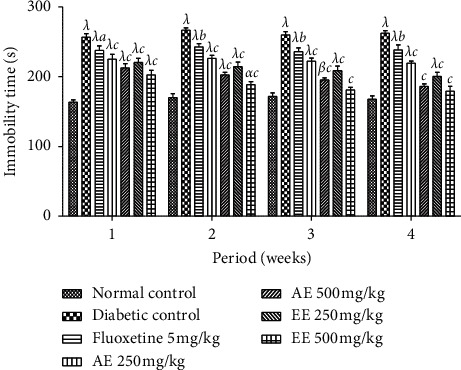
Effects the aqueous (AE) and ethanol (EE) extracts of *Dissotis thollonii* on the immobility time in the force swimming test during 5 min. Values are expressed as mean ± SEM for six animals and analyzed by two-way ANOVA followed by Bonferroni post hoc test. ^*β*^*p* < 0.01, ^*λ*^*p* < 0.001 when compared with the normal control and ^*a*^*p* < 0.05, ^*b*^*p* < 0.01, ^*c*^*p* < 0.001 when compared with the diabetic control; s: seconds.

The animals were forced to swim for 6 minutes and the results are shown in Figure 11. Administration of the extracts and fluoxetine significantly (*p* < 0.001; *p* < 0.05) reduced the duration of immobility in water compared to that of the negative control. The reduction in the duration of immobility was maintained from the first week to the last week of treatment.

**Figure 12:**
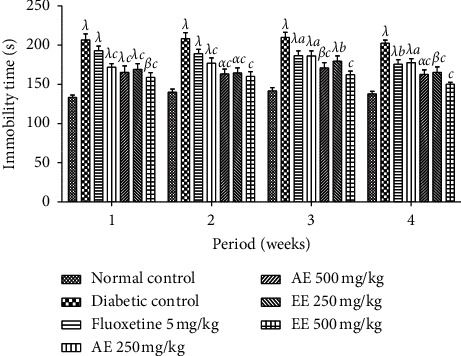
Effects of the aqueous (AE) and ethanol (EE) extracts of *Dissotis thollonii* on the immobility time of mice in the tail suspension test during 5 min. Values are expressed as mean ± SEM for six animals and analyzed by two-way ANOVA followed by Bonferroni post hoc test. ^*α*^*p* < 0.05, ^*β*^*p* < 0.01, ^*λ*^*p* < 0.001 when compared with the normal control and ^*a*^*p* < 0.05, ^*b*^*p* < 0.01, ^*c*^*p* < 0.001 when compared with the diabetic control; s seconds.

In the first week of treatment, the administration of the two extracts significantly (*p* < 0.001) reduced the duration of immobility of the animals suspended by the tail compared to that of the negative control group. This reduction in the duration of immobility remained constant until the end of the treatment. However, the animals in the positive control group significantly (*p* < 0.05) reduced their duration of immobility from the third week compared to that of the negative control group (Figure 12).

**Figure 13:**
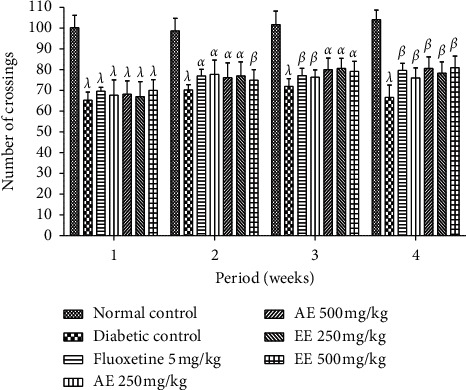
Effects of the aqueous (AE) and ethanol (EE) extracts of *Dissotis thollonii* on the locomotor activity of mice in the open field test during 5 mins. Values are expressed as mean ± SEM for six animals and analyzed by two-way ANOVA followed by Bonferroni post hoc test. ^*α*^*p* < 0.05, ^*β*^*p* < 0.01, ^*λ*^*p* < 0.001 when compared with the normal control.

The variation of the response on the number of passages in the open ground is presented in Figure 13. It appears from this figure that the number of passages in the open ground of all diabetic animals did not change significantly (*p* < 0.001) despite the administration of different treatments compared to the neutral nondiabetic control. However, it should be noted that there was a slight variation between the second week and the last week of treatment.

## 5. Discussion

The present study aimed at evaluating the antihypernociceptive and neuropharmacological effects of the aqueous and ethanol extracts of *D*. *thollonii* leaves on diabetic neuropathy induced by intraperitoneal injection of STZ. This study revealed that the extracts from this plant were capable of inhibiting hyperalgesia (mechanical and chemical) and allodynia (thermal and cold) in mice made diabetic. The extracts did not significantly reduce blood sugar, but improved the body weight of the animals treated compared to the animals of the negative control group. Furthermore, the different extracts also significantly reduced the levels of proinflammatory cytokines (TNF-*α*, IL-1*β*, and IL-6) in the serum and in the sciatic nerve of the treated animals. Similarly, the extracts also significantly stimulated the production of the few growth factors (IGF and NGF) in the sciatic nerve of treated animals.

It is known that one of the most devastating complications of diabetes is diabetic neuropathy which causes the development of thresholds of perception with an increase in abnormal sensations such as hyperalgesia, paraesthesia, allodynia, and pain spontaneous with consequent loss of sensory function in patients [3]. The two characteristics common to chronic pain and normal pain are allodynia (which occurs when a harmless stimulus (light touch) becomes very painful) and hyperalgesia (which occurs when an already painful stimulus causes more intense pain) [37]. Among the animal models of diabetic neuropathy induced in animals, the intravenous injection of streptozotocin (pancreatic toxin of *β*-cells) is the most used [38], with as a consequence development of hyperreactivity and hyperalgesia of C fibers for a period of two to three weeks [39, 40]. In addition, the injection of a single dose of STZ (200 mg/kg) resulted in mice hyperglycemia, significant weight loss, the development of mechanical hyperalgesia, thermal hyperalgesia, and allodynia touch [41–43]. The aqueous and ethanol extracts of the leaves of *D*. *thollonii* significantly inhibited the physical symptoms of diabetic neuropathy caused by the administration of STZ. The results show an inhibition of mechanical allodynia, cold allodynia, thermal hyperalgesia, and chemical hyperalgesia. Furthermore, in a nonsignificant manner, the extracts lowered the blood glucose level and increased the body weight of the diabetic animals treated. These results show that extracts from the leaves of *D*. *thollonii* are rich in secondary metabolites with antihyperalgesic properties. Indeed, phytochemical tests have shown that the extracts of this plant were rich in flavonoids, phenols, anthraquinones, and polyphenols [26], and many compounds belonging to these classes of metabolites have already shown their antihyperalgesic properties [44, 45]. In addition, Nono et al. [25] have isolated from these plant compounds such as betulinic acid and ellagic acid which are compounds with antihyperalgesic properties [46–50].

For chronic pain like neuropathic pain, there is usually an abnormal modulation on several levels, making it difficult to identify the mechanism as well as the choice of the appropriate treatment. After inflammation and/or damage to peripheral or central tissue, the sensitization that occurs in the neurons of the dorsal horn of the spinal cord is the important pathophysiological mechanism that causes secondary hypersensitivity and tactile allodynia [51, 52]. It is known that the reactivity of the sensory nephew system is considerably increased after sensitization by a significant increase in the production of mediators such as nerve growth factors, kinins (bradykinin), proinflammatory cytokines (IL-1*β*, IL-6, and TNF-*α*), amines (histamines), purines (ATP), prostanoids, and ions (potassium and hydrogen) [53–56]. These mediators are capable of acting individually and/or in synergy, to sensitize the nervous system by directly activating the nociceptors to cause painful hypersensitivity [57]. During the development of diabetic neuropathy, neutrophils and macrophages can be recruited and activated by numerous factors (selectins, leukotriene B4, and nerve growth factor), resulting in the significant production of pronociceptive mediators such as lipoxygenase, superoxide, cyclooxygenase, nitric oxide (NO), proinflammatory cytokines (TNF-*α*, IL-1*β*, and IL-6), NGF, prostaglandins, and chemokines [58, 59]. Furthermore, the rates of many growth factors such as NGF are clinically very high in chronic pain conditions (arthritis, diabetic neuropathy, chronic headaches, and cancer pain) [60, 61]. In addition, Mantyh et al. [61] and Dyck et al. [62] have shown that the intradermal injection of NGF in humans or animals results in significant activation and sensitization of nociceptors, thereby maintaining a state of neuropathic pain. The results of this study clearly show that the aqueous and ethanol extracts of the leaves of *D*. *thollonii* significantly inhibited the levels of TNF-*α*, IL-1*β*, and IL-6 in the blood, as well as the levels of TNF-*α*, IL-1*β*, IL-6, NGF, and IGF in the sciatic nerve of the treated animals. In our previous work, these extracts were capable of significantly inhibiting protein denaturation and the activities of COX and 5-LOX, inhibiting the production of ROS, and inhibiting cell proliferation; in addition, these extracts had flavonoids and saponins [26]. The presence in these extracts of compounds such as betulinic acid, *β*-sitosterol, arjunolic acid, and ellagic acid [25] may justify the activities of this plant, since betulinic acid inhibits in vitro the activities of COX and 5-LOX [63, 64], while betulinic acid, *β*-sitosterol, arjunolic acid, and ellagic acid have the ability to inhibit the activation of nuclear transcription factors (NF-kB), resulting in a reduction in the expression of production of proinflammatory cytokines (TNF-*α*, IL-1*β*, and IL-6) and then an inhibition of the activities of iNOS and COX [65, 66].

It is known that in patients with diabetic neuropathy, the presence of a depressed state and/or anxiety is often responsible for worsening pain and also has a very negative impact on the treatment of the patient. Thus, depression and anxiety coexist in people with diabetic neuropathy and can act as mediators and/or complicators of the disease. Furthermore, it has been shown that an antidepressant and/or anxiolytic substance can have a positive effect on the management of pain and the condition of patients suffering from diabetic neuropathy [67, 68]. Since the rates of depression and/or anxiety are very often high in patients with diabetic neuropathy, it is nowadays recommended to systematically screen for depression and/or anxiety in anyone suffering from diabetes [68]. Thus, for a good care of people suffering from diabetic neuropathy, the recommended therapies must be indicated for the relief of pain, but also must fight against depression and anxiety [69, 70]. Elevated labyrinth, dark/light box, social interaction, forced swimming, tail suspension, and locomotion tests are regularly used tests to detect and characterize the antidepressant and anxiolytic activity of many pharmacological substances [71]. Depression may be induced by metabolic disorders of neurotransmitters and/or their monoamines that involve noradrenergic, serotonergic (5-HT), dopaminergic, glutaminergic, and aminergic GABA systems [72]. In addition, it is known that dysfunction of the hypothalamic-pituitary-adrenal axis is involved in the pathophysiology of depression. The continuous activation of this axis is capable of increasing the level of glucocorticoids which consequently alters the function of the hippocampus and neurogenesis [73]. Antidepressants increase the possibility for these monoamines to modulate their function and initiate neurogenesis [74]. The results of our work show that the aqueous and ethanol extracts of the leaves of *D*. *thollonii* have significant antidepressant (raised labyrinth, light/dark box, and social interaction) and anxiolytic (forced swimming, tail suspension, and in the ground) effects. The antidepressant and anxiolytic effects of the extracts could be due to the agonist effect on the GABA/benzodiazepine receptor complex, to an agonist effect on the 5-HT1B receptors and/or to the agonistic activity on the 5-HT1A receptors [75]. This could be justified by the presence in these extracts of ellagic acid which has neuroprotective properties [76] and beta-sitosterol and its derivatives (*β*-sitosterol-3-O-D-glucopyranosyl-6′-mirystate and *β*-sitosterol-3-O-*β*-D-glucopyranoside) which have antidepressant properties by modulating the serotonergic system and the GABA system [77].

## 6. Conclusion

At the end of this study, *D*. *thollonii* has proven its antiallodynic, antihyperalgesic, and antihyperglycemic properties by inhibiting the production of proinflammatory cytokines (TNF-*α*, IL-1*β*, and IL-6) and growth factors (NGF and IGF) and anxiolytic and antidepressant properties. All this confirms the possible effects of extracts of *D*. *thollonii* on neuropathic diabetic pain and neuropharmacological investigation.

## Figures and Tables

**Table 1:** Effects of the aqueous (AE) and ethanol (EE) extracts of *Dissotis thollonii* on blood glucose after injection of streptozotocin in mice.

Treatment	Dose (mg/kg)	Period (weeks)						
		0	1	2	3	4	5	6
Normal control	—	106.67 ± 3.24	104.27 ± 2.18	109.07 ± 5.05	107.67 ± 3.62	104.67 ± 3.93	105.57 ± 2.99	106.40 ± 4.01
Diabetic control	—	104.06 ± 2.82	380.20 ± 8.50^*λ*^	440.03 ± 18.27^*λ*^	446.50 ± 17.88^*λ*^	445.03 ± 20.59^*λ*^	448.23 ± 18.13^*λ*^	456.50 ± 14.27^*λ*^
Pregabalin	30	109.67 ± 4.91	381.00 ± 8.65^*λ*^	434.50 ± 24.89^*λ*^	358.33 ± 13.04^*λc*^	354.33 ± 9.28^*λc*^	357.83 ± 13.19^*λc*^	352.13 ± 11.73^*λc*^
Aqueous extract	250	108.33 ± 0.95	369.33 ± 13.53^*λ*^	438.59 ± 10.54^*λ*^	435.00 ± 4.20^*λ*^	432.62 ± 10.98^*λ*^	428.14 ± 16.35^*λ*^	431.83 ± 14.33^*λ*^
	500	105.50 ± 1.12	361.67 ± 12.33^*λ*^	407.83 ± 13.19^*λ*^	405.50 ± 9.28^*λ*^	404.23 ± 10.47^*λ*^	407.83 ± 12.74^*λ*^	401.83 ± 6.21^*λ*^
Ethanol extract	250	107.00 ± 1.34	376.21 ± 16.17^*λ*^	421.03 ± 12.92^*λ*^	438.33 ± 14.77^*λ*^	429.30 ± 12.72^*λ*^	430.50 ± 16.83^*λ*^	425.17 ± 17.32^*λ*^
	500	106,83 ± 1.47	355.05 ± 9.17^*λ*^	387.19 ± 6.01^*λ*^	388.83 ± 24.59^*λ*^	387.07 ± 19.08^*λ*^	386.17 ± 16.26^*λ*^	378.50 ± 17.67^*λ*^

Values are expressed as mean ± SEM for six animals and analyzed by one-way ANOVA followed by the Tukey post hoc test. ^*λ*^*p* < 0.001 when compared with the normal control and ^*c*^*p* < 0.001 when compared with the diabetic control.

**Table 2:** Effects of the aqueous (AE) and ethanol (EE) extracts of *Dissotis thollonii* on body weight after injection of streptozotocin in mice.

Treatment	Dose (mg/kg)	Period (weeks)						
		0	1	2	3	4	5	6
Normal control	—	23.00 ± 0.88	25.00 ± 1.57	27.33 ± 0.80	29.00 ± 0.82	29.50 ± 1.50	31.00 ± 0.93	31.77 ± 0.99
Diabetic control	—	25.11 ± 1.78	23.22 ± 1.41	21.78 ± 0.24^*α*^	21.01 ± 0.67^*λ*^	19.37 ± 0.44^*λ*^	18.00 ± 0.64^*λ*^	17.33 ± 0.23^*λ*^
Pregabalin	30	26.67 ± 1.52	24.50 ± 0.81	22.17 ± 0.60^*α*^	21.17 ± 0.89^*λ*^	20.67 ± 0.92^*λ*^	19.17 ± 0.79^*λ*^	18.00 ± 0.52^*λ*^
Aqueous extract	250	24.50 ± 1.92	22.17 ± 1.28	21.50 ± 1.29^*β*^	20.50 ± 1.15^*λ*^	19.41 ± 1.43^*λ*^	18.83 ± 1.20^*λ*^	17.09 ± 0.73^*λ*^
	500	25.30 ± 1.69	21.53 ± 0.32	19.83 ± 0.62^*λ*^	18.83 ± 0.60^*λ*^	18.69 ± 0.83^*λ*^	18.00 ± 0.76^*λ*^	16.83 ± 0.70^*λ*^
Ethanol extract	250	25.12 ± 2.03	23.11 ± 0.68	20.83 ± 0.68^*λ*^	19.17 ± 0.73^*λ*^	18.83 ± 0.86^*λ*^	17.83 ± 0.72^*λ*^	16.33 ± 0.56^*λ*^
	500	26.33 ± 1.37	23.28 ± 0.31	21.75 ± 0.47^*λ*^	19.67 ± 0.61^*λ*^	18.17 ± 0.29^*λ*^	17.35 ± 0.40^*λ*^	16.22 ± 0.83^*λ*^

Values are expressed as mean ± SEM for six animals and analyzed by one-way ANOVA followed by the Tukey post hoc test. ^*α*^*p* < 0,05, ^*β*^*p* < 0,01, ^*γ*^*p* < 0,001 when compared with the normal control.

## Data Availability

The data used to support the findings of this study are available from the corresponding author upon request.
